# Unveiling the synergetic benefits of the tunneling technique using stapler tractor in precise resection of lung segments: a retrospective cohort study

**DOI:** 10.3389/fonc.2024.1417871

**Published:** 2024-08-09

**Authors:** Jian Zhu, Cheng-Hao Fu, Liang Chen, Quan Zhu, Shu-Sheng Zhu, Jianan Zheng, Wei Liao, Kun Li, Wei Wen

**Affiliations:** ^1^ Department of Thoracic Cardiovascular Surgery, General Hospital of Central Theater Command of the People’s Liberation Army, Wuhan, China; ^2^ Department of Thoracic Surgery, The First Affiliated Hospital with Nanjing Medical University, Nanjing, China; ^3^ Department of Thoracic Surgery, Taizhou Hospital of Traditional Chinese Medicine, Taizhou, China; ^4^ Department of Anesthesiology, General Hospital of Central Theater Command of the Chinese People’s Liberation Army, Wuhan, China

**Keywords:** lung segmentectomy, tunneling technique, stapler tractor, medical instruments, surgical strategy

## Abstract

**Background:**

Tunneling technique has shown preliminary promise in lung segmentectomy which requires the use of staplers in specific procedures. However, the obstacle when staples pass is the most obvious factor hindering the implementation and development of this technique. This study investigated whether the obstacle of the technology could be addressed by using an innovative self-designed stapler tractor and analyzed the combined and respective advantages of them.

**Methods:**

The clinical data of patients with lung nodules located near anatomical sites with potential tunnel creation treated by segmentectomy were analyzed in this retrospective case-control study. The data were divided into four groups according to four distinct surgical strategies: In Group A, the tunneling technique was performed with a stapler tractor; in Group B, the tunneling technique was performed without a stapler tractor; in Group C, didn’t perform the tunneling technique but using stapler tractor in a normal approach; and in Group D, neither performed the technique nor used the stapler tractor. The general linear data, operation times, intraoperative adverse events, postoperative recovery and complications were compared.

**Results:**

Compared with other groups, Group A exhibited the best surgical outcomes in comprehensive aspects. Separately, the tunnel groups (Group A&B) had better outcomes in the macro implementation of operation, including resection margin, the number of sampled intrapulmonary lymph nodes and resected subsegments, while the staple tractor groups (Group A&C) performed better on details of the procedure, including operation time, conversion to thoracotomy, and intraoperative bleeding (*p* < 0.05). Both of them were beneficial for shorter hospital stay, and the tunnel group was more advantageous.

**Conclusion:**

The tunneling technique is an advanced and beneficial surgical strategy for performing precise resection of lung segments while a stapler tractor can promote and facilitate it as a supplementary instrument. They show more combined benefits in effectively minimizing the occurrence of erroneous injuries and enhancing the operational efficacy.

## Introduction

With the improvement and popularization of thin‐layer computed tomography (CT) scanning technology, an increasing number of lung nodules containing ground-glass opacity (GGO) components have been detected (1, 2). This presents many challenges in the diagnosis and treatment of lung nodules. It is believed that for early lung cancer in the middle or outer third lung field, the long-term effect of sublobectomy (including wedge resection and segmentectomy) is similar to that of lobectomy; additionally, more lung tissue can be preserved while surgical trauma can be effectively reduced (3–6). The JCOG0804 study, JCOG0802 study and CALGB140503 study provide strong evidence for segmentectomy for early peripheral lung cancer with diameter ≤2 cm that demonstrating superior outcomes in terms of postoperative recovery and morbidity compared to lobectomy (7–9).

However, whether intricate segmentectomy is capable of generating multiple or intricate intersegmental planes remains a subject of controversy. If the precise resection of lung segments is aimed, energy-based devices should be used to open the lung parenchyma gradually which can demonstrate the hilus structure of lung vessels and bronchus more clearly (10). This surgical approach is linked to an elevated likelihood of complications, such as prolonged air leakage and bleeding (11, 12). Therefore, the tunneling technique, a technique that can reduce restriction of lung inflation and secure surgical margins from the lesion, has been developed. It’s a technique of segmental plane dissection via staple incisions radially spreading out from the segmental bronchus stump, which is made possible by creating a tunnel passageway to maneuver the stapler’s anvil (13). A few years later, there were only a few anecdotal reports of the application of this technology (14, 15). In terms of the authors’ institutional experience, the tunneling technique must require the use of staplers in specific procedures, but when the staplers pass through the created tunnel, they inevitably meet obstruction and difficulty. This, from our standpoint, is the most obvious factor hindering the implementation and development of the tunneling technique.

Therefore, our study investigated whether the obstacle of the technology could be addressed by using an innovative self-designed stapler tractor and analyzed the combined and respective advantages of them.

## Methods

### Study design and participants

**Figure 1:**
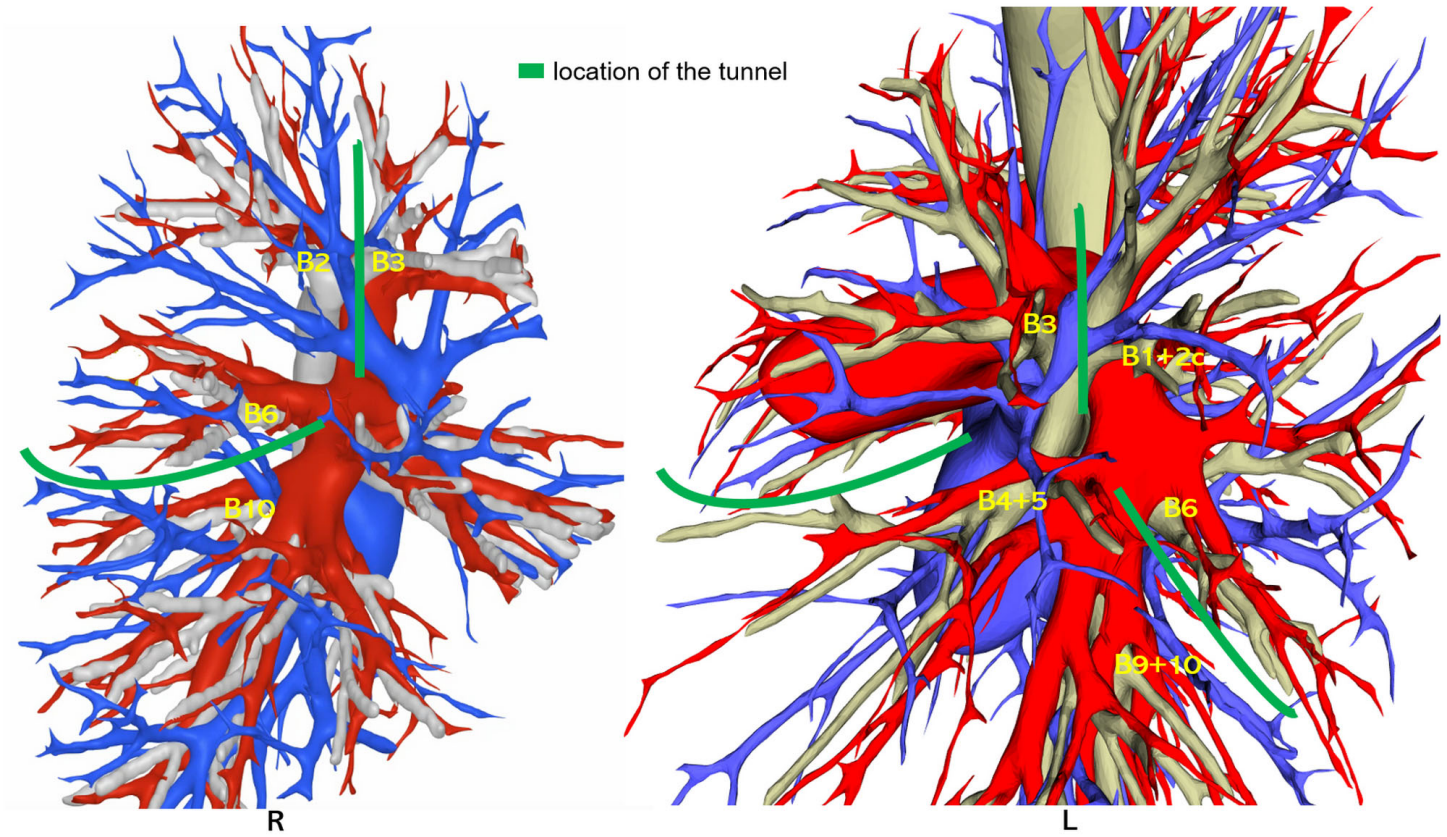
Tunnel location map of the lung segmentectomy procedure.

**Figure 2:**
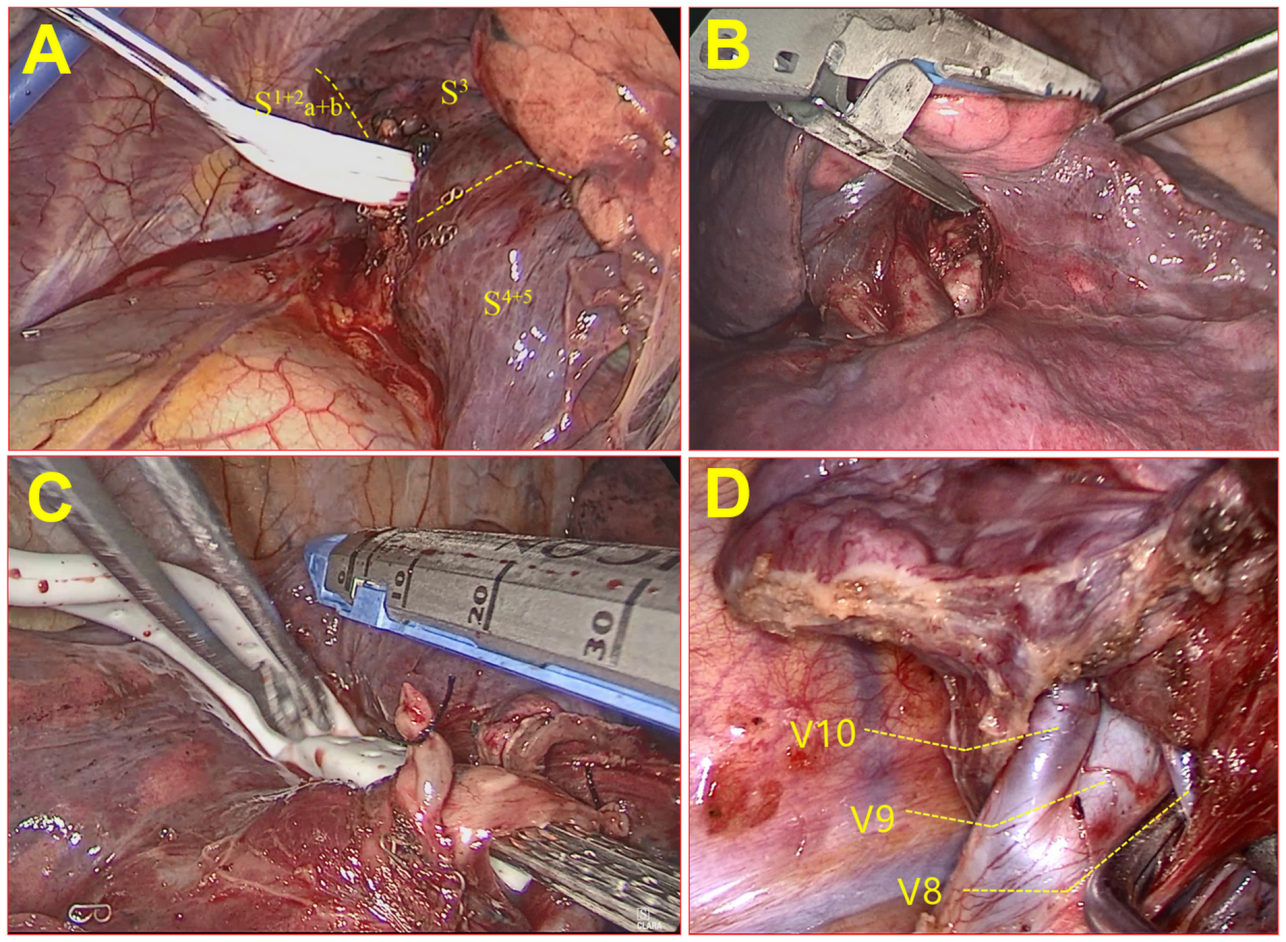
The schematic pictures of the four groups/surgical strategies during the operation. **(A)** Group A, using tunneling technique with stapler tractor group; **(B)** Group B, using tunneling technique without stapler tractor group; **(C)** Group C: using stapler tractor without tunneling technique group; **(D)** Group D, without tunneling technique or stapler tractor group.

Our retrospective case-control study analyzed patients who had lung nodules ≤ 2 cm and located near anatomical sites with potential tunnel creation (see **Figure 1**) and were exclusively treated under the precise resection of lung segments from August 2018 to August 2023 and was conducted by a consistent surgical group. The patients were divided into four groups according to four distinct surgical strategies (see **Figure 2**): In Group A, the tunneling technique was performed with a stapler tractor; in Group B, the tunneling technique was performed without a stapler tractor; in Group C, didn’t perform the tunneling technique but using stapler tractor in a normal approach; and in Group D, neither performed the technique nor used the stapler tractor. The general linear data and surgical outcomes were analyzed, including the duration of operation, bleeding volume, number of sampled intrapulmonary lymph nodes, length of hospital stay, incidence of air leakage, and the counts of resected subsegments. The surgical procedures were conducted exclusively by a consistent surgical group.

**Figure 3:**
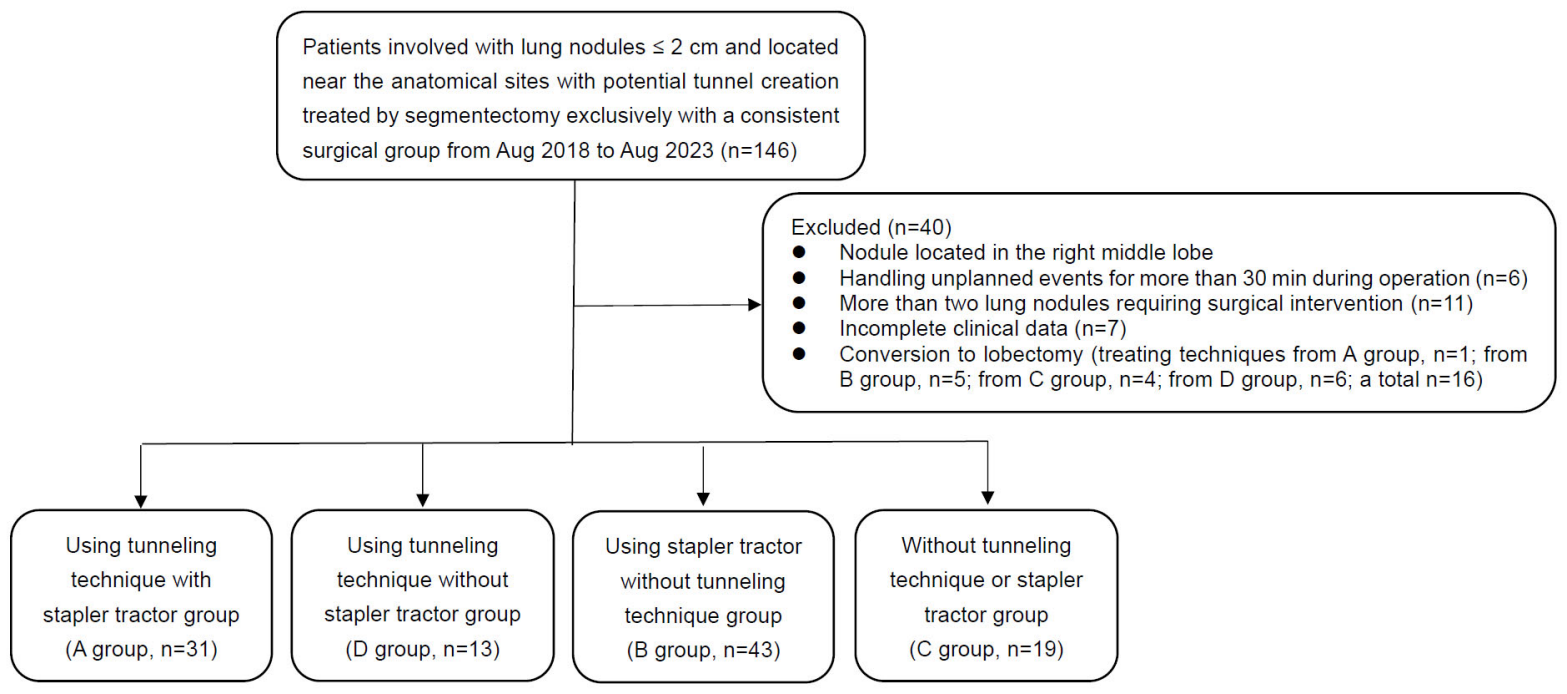
Flowchart of patients. There were 146 patients who met the inclusion and exclusion criteria, and a total of 106 patients were included in this study.

Individuals who had been experiencing unplanned events for more than 30 min during the operation, who had more than two lung nodules requiring surgical intervention, whose clinical data was incomplete, whose nodule located in the right middle lobe (RML) or who had undergone conversion to lobectomy were excluded. The specific patient screening process is illustrated in **Figure 3**.

### Surgical procedures

Prior to the surgical procedure, we underwent three-dimensional (3D) reconstruction to patients aiming at minimally invasive surgery. This reconstruction effectively simulated the segmentation of the lungs and identified lesions, taking into account the tracheal branches arteries, and veins. The labeling of lung nodules facilitated the division of lung segmentation, considering the position and adjacent tissues such as blood vessels and bronchi. Additionally, resection margin spheres were created at a distance of 2 cm from the edge of the lesion. The reconstruction outcomes were evaluated through a comparative analysis with the preoperative CT images and were further deliberated by a panel of highly experienced surgeons holding the titles of associate directors or higher. Precise resection of lung segments is defined as (1) having a safe surgical margin distance more than the maximum diameter of the tumor or 2 cm; (2) dividing and severing the arterial, bronchial, and main venous structures (three are indispensable); (3) using lung subsegment as a unit to reduce the counts of resected subsegments as much as possible.

During video-assisted thoracoscopic surgery (VATS), the segmental lung artery and bronchus of the lesional segment are transected via the mediastinal pleural approach. The lung veins inside the segment are cut off, but the intersegmental lung veins running at the boundary of the segment don’t need to be dissected at this point. The dissecting line of the lung parenchyma was then determined according to the margins from the lesion to the intersegmental line of the lung. A satisfactory surgical margin was determined by preoperative three-dimensional reconstruction and resection margin sphere simulation. Centered on the lesion, the resection portion should include adjacent segments or subsegments.

In all patients, the surgical margins of the surgically resected tissue with lesions were measured. These margins were determined by calculating the shortest distance between the tumor boundary and the line connecting the clip on the pleura with the staples on the bronchial stump, as described previously (16). Our criterion for an acceptable surgical margin distance was that it was equal to or greater than the maximum diameter of the tumor or 2 cm. If this criterion was not met, a lung lobectomy procedure was scheduled to converse to.

### The tunneling technique

**Figure 4:**
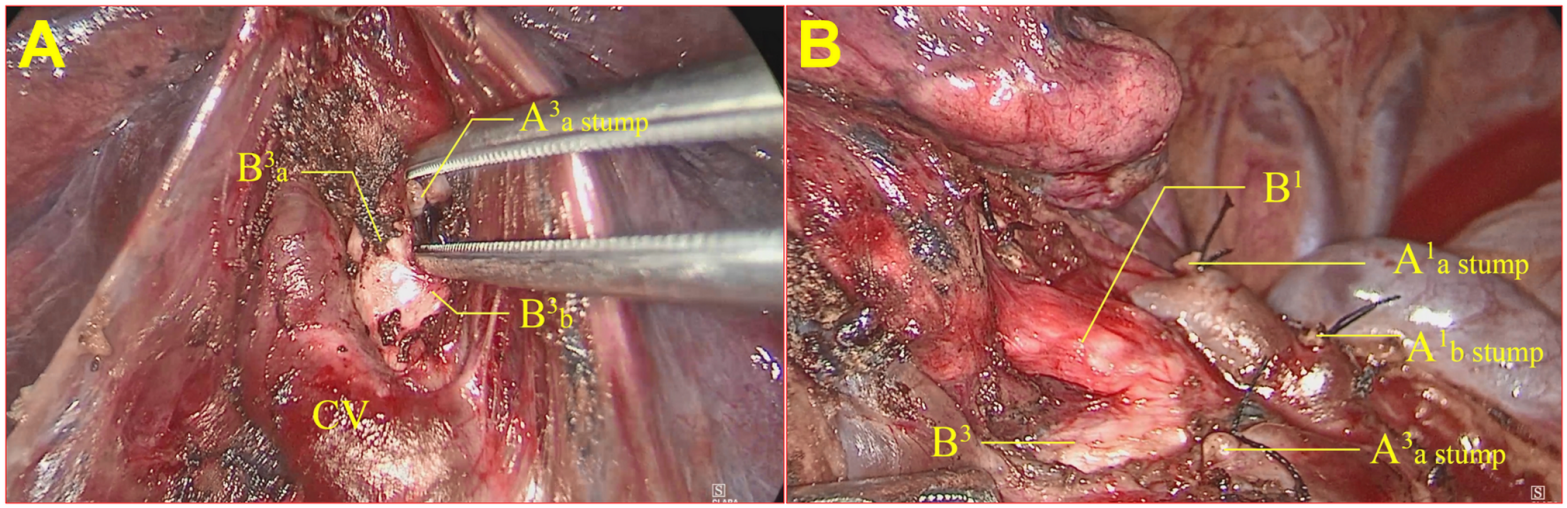
A tunnel was used to establish the incision during resection of the RS1+S3a segment of the right upper lung. **(A)** The entrance hatchway of the tunnel; **(B)** the export hatchway of the tunnel.

The tunneling technique is a technique of segmental plane dissection via staple incisions radially spreading out from the segmental bronchus stump, which is made possible by creating a tunnel passageway to maneuver the stapler’s anvil. Two hatchways of tunneling in the visceral pleura on the dissecting line are created using energy-based devices to enter and exit the tunnel (see **Figure 4**). The structures at the entrance and exit of the tunnel to be severed in both directions should be determined according to 3D reconstruction in the preoperative simulation. During the establishment of the tunnel, it is necessary to check whether there are still unplanned vessels or bronchi inside the tunnel. If these abnormalities are found, there may be errors in the surgical anatomy. Before transecting with staplers, the inflation−deflation method for distinguishing the intersegmental plane is also necessary.

However, the tunnel is formed to be deep and may not be a straight line. Otherwise, there is also an angle with the VATS incision. Therefore, it is very difficult for a stapler to simply pass through.

### The stapler tractor

To solve the above problem, we constructed a novel tractor for staples.

**Figure 5:**
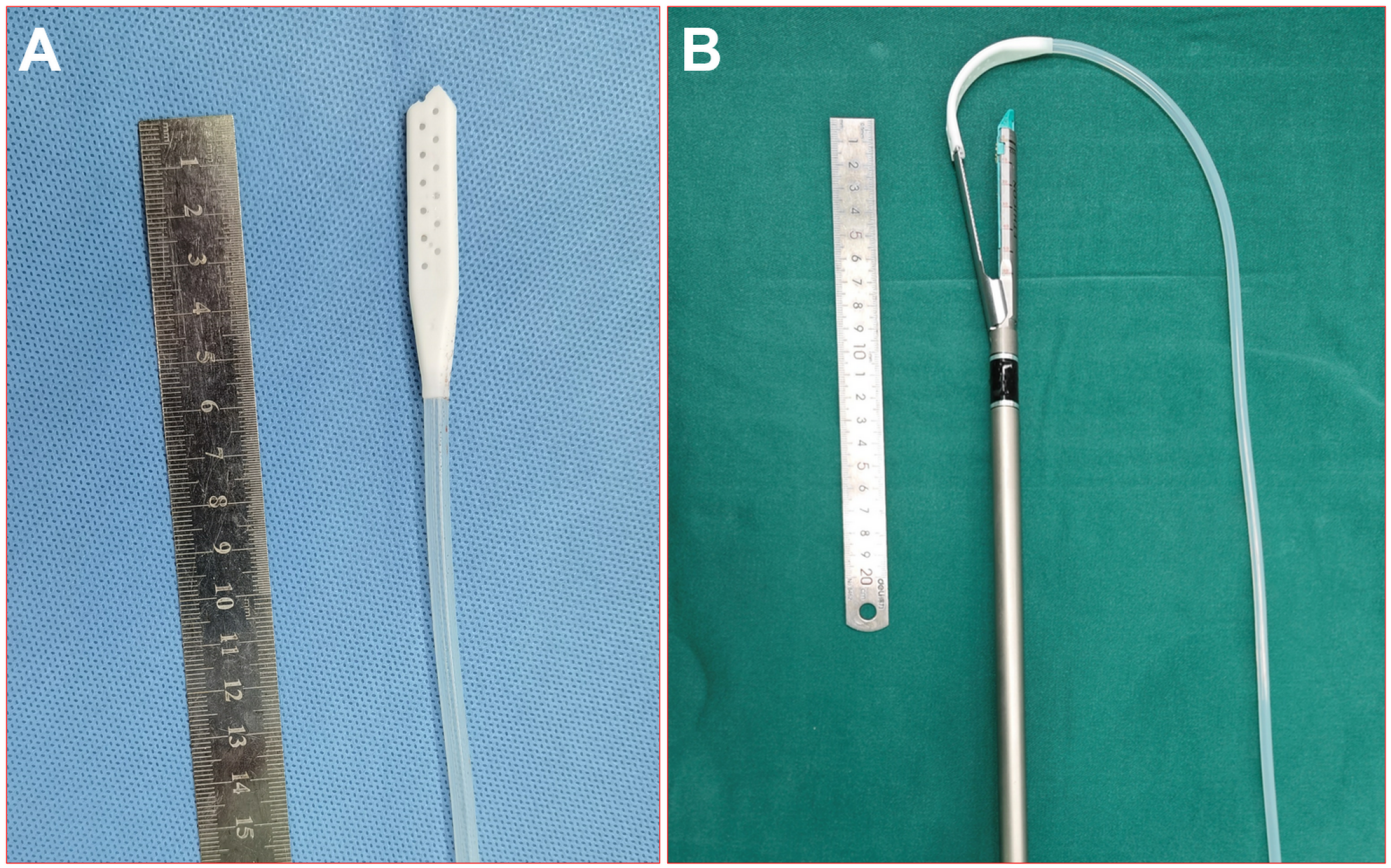
Picture of the real stapler tractor. **(A)** The tractor; **(B)** the tractor worn on the stapler.

The stapler tractor is composed of three main components: a soft silicon flat tube part with a cavity inside (with a length of approximately 5-10 cm), a soft rubber round tube part (with a length of approximately 15-30 cm) with a diameter smaller than the width of the flat tube, and a shifter part that connects the flat tube part and the round tube part (see **Figure 5**). The hollow section cavity of the flat tube was designed to align with the anvil of the stapler (the width was approximately 1.0 cm).

**Figure 6:**
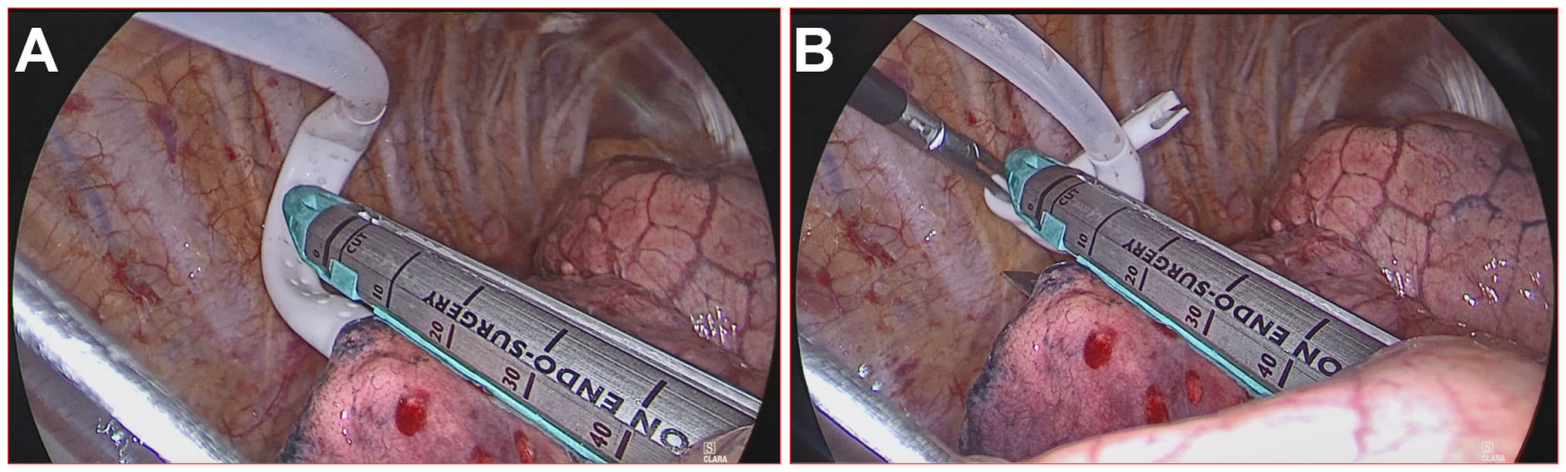
Stapler tractor placement during the operation. **(A)** The worn tractor stapler passes the tunnel with the assistant dragging and guiding; **(B)** the stapler tractor is removed, and the stapler can cut and suture the segment plane.

During the surgical procedure, the anvil of the stapler was inserted through the tunnel using a soft silicon tube guide, and the dissecting line was stapled and transected (group A). During the surgical procedure, the anvil of the stapler was firmly inserted into the cavity of the flat tube. Then, the round tube was gently pulled to guide the stapler to advance toward the target tissue, allowing for sequential passages through the designated safe passage in the following specific order: the round tube, shifter, flat tube, and anvil parts. Subsequently, the stapler was stabilized, the flat tube was extracted, the anvil was exposed, and the stapler tractor was withdrawn from the incisions (see **Figure 6**). The use of staples can better protect the lung parenchyma and vessels and provide a safer and more efficient way to pass through the tunnel. The intersegmental lung veins were closed and divided by stapling. Thus, a standard precise segmentectomy, namely, cone-shaped segmentectomy, proposed by our center, can be achieved (17).

### Data collection and analysis

The data were partitioned into four groups. The eligible medical records were subjected to comparative and analytical examinations including various variables, including age, sex, nodule size (cm), nodule location, nodule solid component, nodule depth ratio, number of intrapulmonary lymph nodes sampled, length of surgical margins (cm), duration of operation (min), intraoperative bleeding (ml), number of required conversion to thoracotomy, counts of resected subsegments, postoperative hospital stay (days), and occurrence of postoperative lung air leakage. The depth ratio method was used for the three symmetrical sectors (3).

The data are presented as the mean ± standard deviation for continuous variables and as absolute numbers and percentages for categorical variables. Categorical variables were assessed using χ2 tests or Fisher’s exact tests as appropriate, while *one-way ANOVA* was conducted for continuous variables, while *t* tests were conducted for continuous variables with independent samples. The nonnormally distributed data were compared between groups by the *U* test. A *p* value ≤0.05 indicated statistical significance. All the statistical analyses were performed using SPSS version 26.0 software.

## Results

### Patient characteristics

Our retrospective case-control study consisted of 106 patients (**Table 1**). The patients had an average age of 54.99 ± 10.18 years included 64 (60.4%) females and 34 (32.1%) smokers. The mean tumor size was 1.08 ± 0.31 cm. A total of 31 patients were included in group A, 13 in group B, 43 in group C and 19 in group D. In total, four lobes and three pathological types of lung nodules from patients who underwent segmentectomy were included. Except for the depth ratio (0.46 ± 0.13 vs 0.45 ± 0.13 vs 0.36 ± 0.06 vs 0.36 ± 0.06, *p* < 0.001), there were no significant differences in the other baseline data among the four groups.

**Table 1:** The baseline characteristics of the four groups.

Variable	Total n=106	Group A n=31	Group B n=43	Group C n=19	Group D n=13	*P* value
Age (year)	54.99 ± 10.18	53.48 ± 11.74	55.98 ± 10.08	55.05 ± 8.94	55.23 ± 8.74	0.7841
Sex (M/F)	42/64	12/19	17/26	7/12	6/7	0.9592
Smoking (no/yes)	72/34	22/9	29/14	13/6	8/5	0.9346
Comorbidity (no/yes) Heart disease COPD Diabetes Hypertension Cerebrovascular	86/20 80/26 87/19 70/36 89/17	26/5 24/7 25/6 21/10 25/6	35/8 32/11 37/6 29/14 37/6	15/4 13/6 15/4 12/7 16/3	10/3 11/2 10/3 8/5 11/2	0.9223 0.7813 0.7927 0.9446 0.9611
Tumor size (cm)	1.08 ± 0.31	1.06 ± 0.31	1.11 ± 0.35	0.99 ± 0.23	1.15 ± 0.32	0.3984
Position RUL RLL LUL LLL	37 19 32 18	14 4 8 5	13 8 13 9	6 5 6 2	4 2 5 2	0.9308
Nodule type pGGO mGGO	41 65	12 19	18 25	7 12	4 9	0.9070
Depth ratio	0.39 ± 0.11	0.46 ± 0.13	0.33 ± 0.06	0.36 ± 0.06	0.45 ± 0.13	<0.001
Pathology results AIS or others noncancerous nodule MIA IAC or others cancerous nodule	48 35 23	15 11 5	19 13 11	10 6 3	4 5 4	0.8458

Group A, using tunneling technique with stapler tractor group; Group B, using stapler tractor without tunneling technique group; Group C, without tunneling technique or stapler tractor group; Group D, using tunneling technique without stapler tractor group; M, male; F, female; COPD, chronic obstructive pulmonary disease; RUL, right upper lung; RLL, right lower lung; LUL, left upper lung; LLL, left lower lung; pGGO, pure ground glass opacity; mGGO, mixed ground glass opacity; AIS, adenocarcinoma *in situ*; MIA, minimally invasive adenocarcinoma; IAC, invasive adenocarcinoma cancer.

### Surgical outcomes among four groups

There were significant differences in multiple surgical outcomes among the four groups.

In all, compared with other groups, Group A (using tunneling technique with stapler tractor) exhibited the best surgical outcomes in comprehensive aspects.

**Figure 7:**
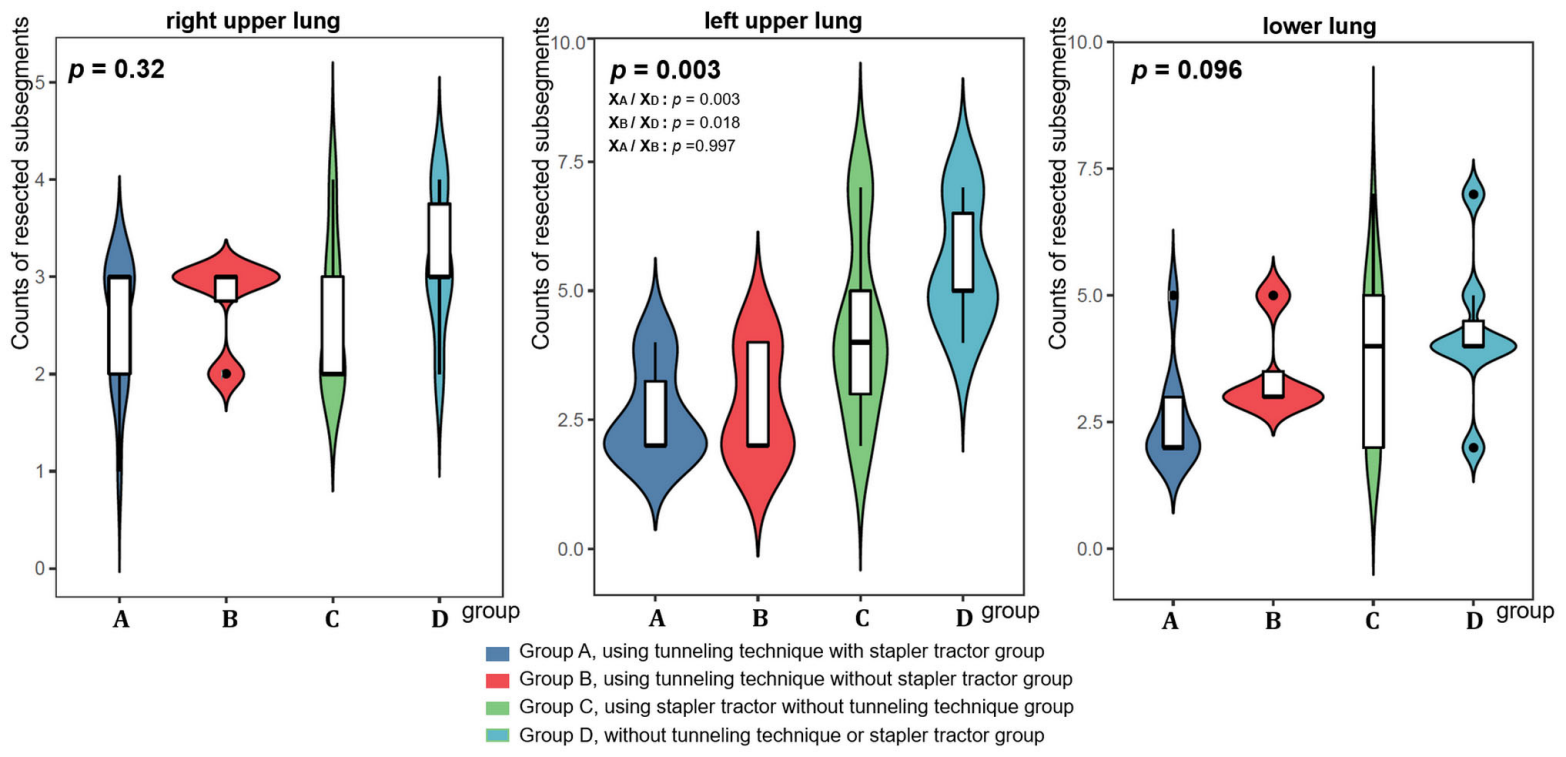
The counts of resected subsegments at different locations on the lungs among the four groups.

Then, the tunneling technique and stapler tractor are considered as two independent factors to divide subgroups and interpret the data information analysis results. The tunnel group (Group A&B), in comparison with the non-tunnel group (Group C&D) had better outcomes in the macro implementation of operation, including longer resection margin, the larger number of sampled intrapulmonary lymph nodes and resected subsegments (M_A+B_/M_C+D_, *p*<0.05, see **Figure 7** and **Table 2**).The staple tractor group(Group A&C), in comparison with the non-staple tractor group (Group B&D) performed better on details of the procedure, including shorter operation time, less conversion to thoracotomy, and less intraoperative bleeding (M_A+C_/M_B+D_, *p*<0.05). Both of them were beneficial for shorter hospital stay, and the tunnel group was more advantageous (M_A+B_/M_C+D_, *p*<0.05, M_A+C_/M_B+D_, *p*<0.05, M_A+B_/M_C+D_<M_A+C_/M_B+D_). However, group B (using tunneling technique without stapler tractor) had longer operation time and the highest probability of air leakage and conversion to thoracotomy which indicated the recommended use of stapler tractor along with tunneling technology.

**Table 2:** Comparison of surgical outcome among the four groups.

Variable	Total n=106	Group A n=31	Group B n=43	Group C n=19	Group D n=13	*P* value
Operative time (min)	133.68 ± 37.46	136.77 ± 44.07	122.79 ± 29.67	129.47 ± 36.62	168.46 ± 23.22	0.0011**^a^**
Surgical margin (cm)	2.23 ± 0.59	2.27 ± 0.37	2.04 ± 0.53	2.35 ± 0.91	2.53 ± 0.44	0.0329^b^
Conversion to thoracotomy (no/yes)	89/17	28/3	38/5	16/3	7/6	0.0298^c^
Intraoperative bleeding (mL)	54.67 ± 30.74	42.42 ± 23.02	50.81 ± 27.08	70.53 ± 36.28	70.53 ± 36.28	0.0009^d^
Postoperative hospital stays (day)	4.43 ± 1.48	3.39 ± 0.62	4.72 ± 1.44	5.37 ± 1.89	4.62 ± 1.04	<0.001^e^
Air leakage (no/yes)	84/22	25/6	37/6	16/3	6/7	0.0314^f^
Intrapulmonary LN sampling counts	2.14 ± 0.86	2.35 ± 0.75	1.88 ± 0.70	1.74 ± 0.65	3.08 ± 1.04	<0.001^g^
Positive LN (no/yes)	102/4	29/2	43/0	19/0	13/2	-
Counts of resected subsegments	3.37 ± 1.45	2.55 ± 0.81	3.65 ± 1.60	4.32 ± 1.49	3.00 ± 0.91	<0.001^h^

Group A, using tunneling technique with stapler tractor group; Group B, using stapler tractor without tunneling technique group; Group C, without tunneling technique or stapler tractor group; Group D, using tunneling technique without stapler tractor group. LN, lymph node;

^a^X_A_/X_D_: *p*=0.0369<0.05, X_A_/X_B_: *p*=0.3345 >0.05, M_A+D_/M_B+C_: *p*=0.001<0.05, M_A+B_/M_C+D_: *p*=0.0238<0.05;

^b^X_A_/X_D_: *p*=0.5299>0.05, X_A_/X_B_: *p*=0.3267 >0.05, M_A+D_/M_B+C_: *p*=0.005<0.05, M_A+B_/M_C+D_: *p*=0.0665 >0.05;

^c^X_A_/X_D_: *p*=0.0121<0.05, X_A_/X_B_: *p*=1 >0.05, M_A+D_/M_B+C_: *p*=0.2965>0.05, M_A+B_/M_C+D_: *p*=0.0257 <0.05;

^d^X_A_/X_D_: *p*=0.0081<0.05, X_A_/X_B_: *p*=0.6046 >0.05, M_A+D_/M_B+C_: *p*=0.3181>0.05, M_A+B_/M_C+D_: *p*<0.001;

^e^X_A_/X_D_: *p*=0.0002<0.05, X_A_/X_B_: *p*<0.001, M_A+D_/M_B+C_: *p*=0.0001<0.05, M_A+B_/M_C+D_: *p*=0.0034 <0.05;

^f^X_A_/X_D_: *p*=0.033<0.05, X_A_/X_B_: *p*=0.534>0.05, M_A+D_/M_B+C_: *p*=0.0601>0.05, M_A+B_/M_C+D_: *p*=0.0798>0.05;

^g^X_A_/X_D_: *p*<0.001, X_A_/X_B_: *p*=0.0455<0.05, M_A+D_/M_B+C_: *p*<0.001, M_A+B_/M_C+D_: *p*=0.5373>0.05;

^h^X_A_/X_D_: *p*=0.5306>0.05, X_A_/X_B_: *p*=0.0044<0.05, M_A+D_/M_B+C_: *p*<0.001, M_A+B_/M_C+D_: *p*=0.0248<0.05.

## Discussion

The improvement and popularization of thin-layer CT scans, the extensive implementation of artificial intelligence and three-dimensional reconstruction techniques have led to the identification of a growing number of small lung nodules (18, 19). Surgical intervention encompassing lobectomy, segmentectomy, and wedge resection constitutes the primary treatment approach for small lung nodules (20, 21). Recently, segmentectomy has emerged as a prevalent surgical procedure (22, 23). Segmentectomy, involving the removal of a single lung segment or specific lung segments, such as the superior segment, lingual segment, and other simple lung segments, has been widely performed by many county-level hospitals in China.

The concept of precise lung segments resection proposed by our center further expands the boundaries and possibilities of sublobectomy. Meanwhile, the tunneling technique has broken through the anatomical barriers of multiple segments to a certain extent. But how to underdo precise lung segments resection on lung nodules near complex anatomical sites with potential tunnel creation have become a technical bottleneck. Surgical instruments for increasing the success rate of the tunneling technique are worth exploring. Among them, how to handle the obstacle of stapler passage appears to be the he most obvious one. Hence, we constructed a novel tractor for staples. This study findings indicate that the depth ratio of nodules in the tunnel group (Group A&B) was larger and the lesion was closer to the deep lung parenchyma. It was more difficult for these two groups to implement precise segments resection by conventional approaches, which suggested the aid and significance of the tunnel technology for surgery. Group C and group D (groups without tunneling technique) had nodules closer to the outer 1/3 field and were more likely to undergo an enlarged wedge resection.

In this study, the tunnel and the stapler tractor respectively showed great surgical outcomes. Generally, group A (using tunneling technique with stapler tractor) showed the best combined outcome, suggesting the great advantage of the combination in precision segmentectomy. The poor outcome of group B (tunneling technique without stapler tractor) in some certain terms like operation time/air leakage further suggested that it is best to use the stapler tractor in combination with the tunnel. This is also consistent with the fact that the tunnel is long and deep, and there is a fixed angle deviation. Without the use of tractor, the tunneling technique is difficult to achieve on the one hand, and there must be unpredictable intraoperative complications on the other hand.

The tunnel where the precise resection of lung segments is performed is usually of a certain length, either in the upper or lower lobes. In addition, there is an angle between the incisions in VATS. It is almost impossible to sever through the intersegmental plane of the tunnel with a single stapler (24). It is easy to imagine that without the guidance of the tractor, the front end of the stapler would be in the blind zone inside the tunnel, providing an uncontrolled condition for mis-resected tissues. Improper dissection of the intersegmental plane is one of the most important factors leading to intraoperative and postoperative complications (25). One detail that must be mentioned here is that the stapler can sever the intersegmental plane without tractor being removed from itself. But when it is used to sever blood vessels, the tractor must be removed from the tube. This is because the lung tissue is thick, and nothing will be affected without removing the tractor. However, when dealing with segmental vessels, without removing the tractor, the small blood vessels won’t be sutured tightly, and the blood will certainly be sprayed. This surgical instrument evidently mitigates the occurrence of intraoperative hemorrhaging and pulmonary air leakage in precise resection of lung segments through three factors. First, the stapler tractor is constructed from a pliable silicone material, allowing for gentle passage through the tunnel and forming a physically closed loop that safeguards the adjacent tissues against harm. Second, the use of a stapler tractor within the tunnel necessitates minimal space, thereby optimizing the operational efficiency of the unobstructed ingress and egress of the tunnel. Third, the robustness of the stapler’s anvil poses a potential risk of lung tissue damage. However, the application of a soft silicone flat tube effectively safeguards both the lung tissue and blood vessels.

In addition, we would like to emphasize the superiority of the tunneling technique. At present, the clinical application of anatomical sublobectomy in thoracic surgery has been gradually promoted. The key to a successful precise resection of lung segments is to distinguish the anatomical structure of lung vessels and bronchi by three-dimensional computed tomographic bronchography and angiography (3D-CTBA) (3, 10). Therefore, the majority of surgeons specializing in VATS precision resection of lung segments have received training to achieve a certain level of proficiency in managing central vessels, dissecting incomplete fissures to locate the pulmonary artery (PA), excising N1 nodes along the artery and bronchus, identifying anatomical variations, and subsequently carrying out the division of bronchovascular structures. To obtain an accurate segmentation, separation of the lung fissures first and identification of the vessels and bronchus later are two of the most practical techniques during the early phase of the learning curve for segmentectomy, especially for surgeons who prefer the fissure approach. However, each patient must have more or less variation in the lung fissures. Any variation in the architecture of the fissures, such as an incomplete fissure, an absent fissure, an accessory fissure or a pseudoazygos fissure may change the lobar or segmental patterns of the lung, affecting the accuracy of the operation. Thoracic surgeons should understand that distinct gaps predicatively exist among the bronchi, arteries, and veins within the various lung lobes, even in cases where an interlobar fissure is not fully formed.

Consequently, how to comprehend the tunneling technique and create a tunnel during surgical thoracic operation actually correspond to three stages. First, when performing lobectomy on a patient with an incomplete interlobar fissure, the tunnel technique is utilized if the interlobar fissure approach is still preferred (26). Second, distinct gaps can be observed between the superior segment and the basal segment, as well as between the lingular segment and the intrinsic segment, even in the absence of a fissure. Additionally, the spaces between RS3 and RS1+RS2, LS3 and LS1 + 2, and S9+S10 and S7+S8/S7 + 8 may also be anatomically separated by tunneling techniques. From this point of view, we can better understand the advantages of using a stapler tractor in tunneling technology.

### Limitations

This was not a prospective randomized controlled study because of the necessity of reviewing the specifics of surgical video replays. To mitigate confounding factors, the study exclusively enrolled patients who underwent surgeries at a single center and who underwent a single operation. Consequently, the generalizability and applicability of the study’s findings may be subject to some deviation.

## Conclusions

In conclusion, the use of a stapler tractor in the tunneling technique is a safe and efficient approach for performing precision resection of lung segments, and it can reduce the count of resected subsegments. The tractor serves as a supplementary instrument in the tunneling technique, effectively minimizing the occurrence of erroneous injuries and enhancing the operational efficacy. This technique presents a potential avenue for overcoming the challenges encountered in intricate anatomical regions where the precise resection of lung segments is arduous. Furthermore, patients experienced expedited postoperative recuperation. Significantly, the use of the stapler tractor in the tunneling technique facilitates clearer visualization of the hilus structure, which includes the lung vessels and bronchus, thereby enhancing the success rate of surgery for beginner thoracic surgeons.

## Data Availability

The original contributions presented in the study are included in the article, further inquiries can be directed to the corresponding authors.
